# Role of ICU nurses in the confrontation of post-traumatic stress disorder in relatives of ICU patients in a general hospital of Athens, Greece

**DOI:** 10.1186/cc11095

**Published:** 2012-03-20

**Authors:** M Kourti, T Katostaras, G Kallergis, E Christofilou, I Floros, G Fildisis

**Affiliations:** 1 Laiko General Hospital, Athens, Greece; 2 University of Athens, Faculty of Nursing, Athens, Greece

## Introduction

This study was planned to assess post-traumatic stress disorder (PTSD) in relatives of ICU patients and to evaluate the role of ICU nurses in the confrontation of these symptoms.

## Methods

The Impact of Event Scale - Revised (IES-R) was translated and distributed to the family members of patients that were hospitalized in the ICU from August 2008 to September 2010. Two measurements took place: the first one 7 to 10 days from the admission of the patient to the ICU and the second one (to the same relative) after 15 to 20 days from the admission. The maximum IES-R score is 88 (0 to 4 for each one of the 22 questions that constitute the scale). Scores over 33 were interpreted as severe cases of PTSD. Patients' health condition was evaluated with the APACHE II score before each measurement.

## Results

From the first measurement it occurred that 66.7% of the relatives faced severe symptoms of PTSD (scores >33) and from the second measurement it occurred that 70% of family members were identified as cases of severe stress symptoms too. No correlation was found between these symptoms and APACHE II score (*P *> 0.05), indicating that such symptoms exist in family members during the whole patient's stay in the ICU, regardless of the seriousness of the patient's condition.

## Conclusion

Relatives of ICU patients seem to suffer from symptoms of PTSD. Nurses who work in the ICU, and have direct and longer contact with patients and relatives too, need to recognize, evaluate and minimize these symptoms in order that further disorders and damage to the relatives' mental health be prevented.
